# Deducing the Impact of Physical Activity, Sedentary Behavior, and Physical Performance on Cognitive Function in Healthy Older Adults

**DOI:** 10.3389/fnagi.2021.777490

**Published:** 2022-01-05

**Authors:** Sina Gerten, Tobias Engeroff, Johannes Fleckenstein, Eszter Füzéki, Silke Matura, Ulrich Pilatus, Lutz Vogt, Johannes Pantel, Winfried Banzer

**Affiliations:** ^1^Faculty of Psychology and Sport Science, Department of Sports Medicine, Institute of Sports Sciences, Goethe University, Frankfurt, Germany; ^2^Faculty of Psychology and Sport Science, Neurocognition and Action – Biomechanics Research Group, Department of Sports Sciences, Bielefeld University, Bielefeld, Germany; ^3^Center of Excellence “Cognitive Interaction Technology”, Bielefeld University, Bielefeld, Germany; ^4^Division Health and Performance, Institute of Occupational, Social and Environmental Medicine, Faculty 16, Goethe University Frankfurt, Frankfurt, Germany; ^5^Institute of General Practice, Goethe University, Frankfurt, Germany; ^6^Department of Psychiatry, Psychosomatic Medicine and Psychotherapy, Goethe University, Frankfurt, Germany; ^7^Institute of Neuroradiology, Goethe University Hospital, Frankfurt, Germany

**Keywords:** cognition, cardiorespiratory fitness, dementia, older adults, exercise

## Abstract

**Objectives:** Participating in physical activity and maintaining physical performance as well as reducing sedentary behavior are discussed to be beneficially associated with cognitive function in older adults. The purpose of this cross-sectional analysis was to differentiate the relevance of objectively measured physical activity, physical performance, and sedentary behavior on cognitive function in healthy older adults (*n* = 56, age = 76 ± 7 yrs, gender = 30 female).

**Methods:** Accelerometer based physical activity and sedentary behavior were analyzed as minutes per week spent sedentary and physically active with light or moderate to vigorous intensity. Participants' physical performance was assessed via cardiopulmonary exercise testing and analyzed as maximal workload and heart rate, heart rate reserve and peak oxygen uptake. The assessment of cognitive function included working memory, attention, executive function, and verbal memory. Data was analyzed with Spearman and partial correlations. Trial registration: NCT02343029.

**Results:** Light physical activity was moderately associated with executive function (*r* = −0.339, *p* = 0.015). Attention was significantly associated with maximal workload (*r* = −0.286, *p* = 0.042) and peak oxygen uptake (*r* = −0.337, *p* = 0.015). Working memory was associated with maximal workload (*r* = 0.329, *p* = 0.017).

**Conclusion:** Whereas a broad range of cognitive function were beneficially linked to physical performance, light intensity activities in particular showed an impact on executive function. Our research underlines the need to separate the impact of physical performance and physical activity on cognitive function and highlights the relevance of light physical activity.

## Introduction

Physical activity (PA) is discussed to alleviate age related cognitive decline and to be capable to improve cognitive function (CF) in older adults (Colcombe and Kramer, 2003; Northey et al., 2017; Ströhlein et al., 2017). However, evidence concerning this association is equivocal and some systematic reviews have failed to confirm a relationship between PA and cognitive functioning (Angevaren et al., 2008; Young et al., 2015). These reviews may indicate that the impact of PA could be specific for cognitive domains or could be influenced by the intensity or type of PA.

Cross-sectional studies have reported that objectively measured PA is related to executive function (Barnes et al., 2008; Wilbur et al., 2012; Kerr et al., 2013; Johnson et al., 2016) and memory (Zhu et al., 2015). Taking PA intensity into account, some studies showed a beneficial effect of light intensity physical activity (LIPA) (Wilbur et al., 2012; Zhu et al., 2015; Johnson et al., 2016), whereas others reported an influence of moderate to vigorous intensity physical activity (MVPA) (Wilbur et al., 2012; Kerr et al., 2013; Zhu et al., 2015). Steinberg et al. (2015) found a beneficial association of everyday activities like watering houseplants and walking while shopping. Since many older people have limited abilities to participate in moderate or high intensity activities, it is of utmost importance to further analyze the health enhancing potential of LIPA for this population.

Whereas, older recommendations solely featured moderate to vigorous intensity physical activities, the new WHO guidelines on health enhancing physical activity recommend replacing sedentary behavior (SB) with any intensity of PA including LIPA (Bull et al., 2020). SB is defined as any behavior that involves energy expenditure at the level of 1.0–1.5 metabolic equivalent units (METs) in a reclined or sitting position (Pate et al., 2008). Likewise to the increasing interest in the connection between PA and CF, there is a growing research area in SB (Voss et al., 2014). Recent reviews conclude that SB might be associated with a decrease in cognitive performance (e.g., attention) (Falck et al., 2017; Olanrewaju et al., 2020). However, to date there is limited research based on objective data on the relationship between SB and cognitive outcomes (Rhodes et al., 2012; Olanrewaju et al., 2020).

One of the most relevant mechanisms for PA and SB effects on CF is the alteration of cardiorespiratory fitness (CRF). Studies were able to show an association between CRF, CF and underlying brain metabolic adaptations in older adults (Newson and Kemps, 2006; Erickson et al., 2009, 2012; Engeroff et al., 2018, 2019; Rowley et al., 2020). Rowley et al. (2020) could show microstructural changes in the motor cortex which where correlated to the oxygen consumption at the first ventilatory threshold after endurance training in older adults. Engeroff et al. (2018) found that brain derived neurotrophic factor (BDNF) was detrimentally associated with SB but beneficially related to accelerometer total activity counts and MVPA. The release of BDNF is considered as a key mechanism for adaptive responses of the brain to metabolic challenges (Marosi and Mattson, 2014) and is discussed as an essential mediator for PA induced changes in brain plasticity (Huang et al., 2014). Erickson et al. (2009, 2012) showed a beneficial relationship of CRF with N-Acetylaspartic acid (NAA), hippocampal volume and spatial working memory in older adults. NAA is a metabolite in the nervous system (Nadler and Cooper, 1972), which exists mostly in cell bodies of neurons (Moffett et al., 2007). Higher concentrations of NAA could be associated with neuronal viability and vascularization (Erickson et al., 2012). Across a wider age span, Newson and Kemps (2006) compared the relationship between CRF (VO_2max_) and domain specific CF in young (18–30 years), young-old (65–74 years), middle-old (75–84 years) and old-old adults (85–92 years). The authors found a beneficial association between CRF and attention and working memory but no significant association between CRF and executive function and memory. Most studies only examined the effects of aerobic capacity assessed as VO_2max_ and ignored outcomes for overall capacity such as maximal heart rate (HR), heart rate reserve (HRR) or maximal workload. These outcomes can help to gain a better understanding about the effect of physical performance (PP) on CF and should therefore be used for the analysis in future studies.

To date no study has investigated the influence of physical activity intensity and physical fitness on CF by using gold standard measures such as accelerometry, cardiopulmonary exercise testing and a validated cognitive assessment including multiple relevant domains in healthy older adults. The present study aimed at analyzing the association of domain specific CF (attention and psychomotor speed, executive function, working memory and verbal memory) with accelerometer-based PA and SB as well as with objectively measured PP in healthy older adults.

## Materials and Methods

### Study Design

The research was conducted as part of the SMART study (NCT02343029; ClinicalTrials.gov) (Fleckenstein et al., 2015). Results of the randomized controlled trial have been reported elsewhere (Matura et al., 2017). The present study used baseline cross-sectional data to investigate the association of PP, PA, and SB with CF in older age. The study protocol was reviewed and approved by the ethics commission of the medical faculty of Goethe University Frankfurt (reference 107/13) and in agreement with the Declaration of Helsinki (Version Fortaleza 2012).

### Participants and Setting

Participants were recruited in three residential facilities and via a local newspaper article published by the university press agency. Healthy older adults (≥65 years old) who fulfilled the inclusion criteria for details see (Fleckenstein et al., 2015) were included. Participants who were willing to take part in the study had to give their written informed consent and were tested on two separate days, within 1 week on cognitive performance (day 1) and PP and current PA data (day 2).

### Cognitive Function

CF was assessed in four domains: attention, psychomotor speed, executive function, working memory and verbal memory. Attention and psychomotor speed was evaluated via Trail Making Test (TMT) A (Tombaugh, 2004). Executive function was assessed via Trail Making Test B (Tombaugh, 2004) and the Stroop Color-Word Test (van der Elst et al., 2006). TMT A is assumed to require mainly visuoperceptual abilities, TMT B is measuring working memory and task-switching ability, whereas TMT B times minus A times (TMT B-A) reflects a rather separated indicator of executive control abilities (Sánchez-Cubillo et al., 2009; Kerr et al., 2013). The Stroop Color-Word Test was analyzed as mean time for word reading (Stroop I), color naming (Stroop II), color-word interference (Stroop III) and as Stroop Interference Score (Interference score = Stroop III – [(Stroop I + Stroop II)/2]). The main outcome of the Stroop Test to measure executive function is the Stroop Interference Score, which quantifies the inhibition of a habitual response while simultaneously processing two stimulus features that affect the same stimulus (Valentijn et al., 2005; van der Elst et al., 2006). Working memory was assessed via digit span forward and backward tests. The difference between the two versions of digit span is that digit span forward can be almost completely processed by the operations of the phonological loop with minimal support of the central executive, whereas digit span backward is handled mostly by the inclusion of the central executive (Grégoire and van der Linden, 1997). Memory was assessed as verbal declarative memory (Morris et al., 1989) with the Verbal Learning and Memory Test (VLMT) (Helmstaedter et al., 2001). Crystallized intelligence was assessed via a validated multiple choice word test (MWT-B) and analyzed as intelligence quotient (IQ) (Lehrl et al., 1995). The Mini Mental State Examination (MMSE) was used to screen for signs of dementia and cognitive impairment with a cut-off value of 27 (Maximum Score = 30) (Folstein et al., 1983).

### Anthropometry, Physical Performance, and Current Physical Activity

The data for anthropometry, PP and current PA were assessed on the same day. Body mass in kilograms was measured using a digital scale to the nearest 0.1 kg, height in centimeters was determined to the nearest 1 cm using a stadiometer and body mass index (BMI) was calculated (BMI; kg/m^2^). Participants' physical performance was assessed via cardiopulmonary exercise testing using a step incremental protocol on a bicycle ergometer (“custo control,” customed GmbH, Munich, Germany). The following physical performance measures were used: (1) maximal workload (in Watt, W) (2) maximal relative workload (in Watt to bodyweight ratio, W/kg), (3) maximal HR (in beats per minute, bpm) (4) heart rate reserve (HRR, calculated as maximal heart rate minus resting heart rate in bpm) and (5) peak oxygen uptake (VO_2peak_ in milliliters per kilogram bodyweight per minute, ml/kg/min). The exercise testing protocol started with zero Watt and increased the work load every 3 min by 25 Watts. Current PA was assessed as counts via accelerometry (GT1M, ActiGraph, Pensacola FL, USA) and analyzed as minutes per week spent in sedentary (SB ≤ 99 counts per minute, cpm), light (LIPA 100−1,951 cpm), moderate to vigorous PA (MVPA ≥ 1,952 cpm) and total activity counts (TAC) (Freedson et al., 1998). Participants were asked to wear accelerometers for 7 consecutive days and data from 4 valid test days (at least 10 h wear time) were used for analysis.

### Statistics

Study sample characteristics are presented as mean ± standard deviation. As data were not normally distributed, Spearman correlations were used to evaluate the association between PA, PP, and CF. In cases of significance a second analysis was used to analyze the impact of confounders. Based on reports of earlier studies (van der Elst et al., 2006), potential confounding effects of sex, age, BMI, crystallized intelligence and education were assessed. Sex, age, BMI, and education had a significant influence and were applied as covariates for partial correlation analysis. The second correlation analysis was applied solely for parameters of CF showing a significant relation to PA or PP data. The level of significance was *p* < 0.05. The effect size is given as correlation coefficient (*r*) with very high (*r* = 1.0), high (*r* = >0.70–0.99), moderate (*r* = >0.4–0.69), small (*r* = >0.0–0.39) and no (*r* = 0.0) correlation (Bös et al., 2004). Data analysis was performed as available-case analysis with the SPSS statistical software system, version 24.0 (SPSS Inc., Chicago, IL, USA).

## Results

### Study Sample Characteristics and Cognitive Performance

The datasets of fifty-six cognitively healthy older adults were included in this analysis (age: 64–90 yrs, 30 female). Descriptive data of study sample characteristics, PA, PP and CF are shown in Table 1.

**Table 1:** Descriptive data of study sample characteristics, current physical activity, physical performance, and cognitive performance.

***n*** **=** **56**		**Mean ± Standard deviation**
**Study sample characteristics**		
Age (years)		76 ± 7
Body size (cm)		167.8 ± 9.8
Body weight (kg)		73.3 ± 12.9
Body mass index (kg/m^2^)		25.9 ± 3.6
Education (years)		12.6 ± 3.5
MMSE (points)		29.0 ± 1.1
MWT-B (IQ)		124.8 ± 10.7
**Current physical activity**		
Total activity counts (min/day)		255.4 ± 156.7
Moderate to vigorous physical activity (min/week)		210.9 ± 198.8
Light physical activity (min/week)		1284.2 ± 320.4
Sedentary time (min/week)		4324.8 ± 619.1
**Physical performance**		
Peak oxygen uptake (ml/kg/min)		21.7 ± 5.9
Maximal workload (Watts)		98.1 ± 38.2
Relative maximal workload (Watts/kg)		1.33 ± 0.45
Maximal heart rate (bpm)		141.9 ± 23.3
Heart Rate Reserve (bpm)		69.8 ± 22.1
**Cognitive performance**		
Attention and psychomotor speed	Trail making test Part A (sec)	46.7 ± 18.3
Executive function	Trail making test Part B (sec)	106.2 ± 41.2
	TMT-B/TMT-A (sec)	2.38 ± 0.74
	Stroop interference Score (sec)	49.2 ± 16.8
Working memory	Digit span forward (points)	8.7 ± 2.1
	Digit span backward (points)	6.7 ± 2.3
Verbal memory	VLMT Trial 1—Trial 5 (items)	44.3 ± 9.8
	VLMT Trial 5—Trial 7 (items)	2.7 ± 2.1
	VLMT recognition—mistakes (items)	10.1 ± 4.1

*Number of participants (n) and mean ± standard deviation of study sample characteristics: Age in years, body size in centimeters (cm), body weight in kilograms (kg), body mass index (BMI) in kilograms per square meter (kg/m^2^), education level in years, mini mental state examination (MMSE) in points and crystallized intelligence assessed via MWTB as intelligence quotient (IQ); Current physical activity: total activity counts (TAC, in min/day), moderate to vigorous physical activity (MVPA, in min/week), light intensity physical activity (LIPA, in min/week) and time spend sedentary (SB, in min/week); Physical performance: of peak oxygen uptake (ml/kg/min), maximal workload (Watt), relative maximal workload (Watt to bodyweight ratio), maximal heart rate (HR, bpm) and heart rate reserve (HRR, maximal heart rate—resting heart rate, bpm) and cognitive performance: attention and psychomotor speed (Trail Making Test (TMT) Part A in sec), executive function (TMT Part B in sec, ratio score B/A in sec, Stroop Interference Score in sec), working memory (digit span forward and backward) in points and verbal memory (Verbal Learning and Memory Test, VLMT: Total Learning Performance (Σ Trial 1–5) in items, loss after delay (Trial 5–7) in items, Correct Recognition Performance (recognition—mistakes) in items)*.

### Relationship Between Cognitive Function and Current Physical Activity

Spearman correlation revealed a positive association (*r* = −0.359, *p* = 0.007) between executive control function (TMT ratio score B/A) and LIPA. Verbal Memory (VLMT *Σ* Trial 1–5) was positively associated with TAC (*r* = 0.294, *p* = 0.028) and with LIPA (*r* = 0.767, *p* = 0.047). Furthermore, verbal memory (VLMT *Σ* Trial 1–5) showed a negative correlation with SB. Further analysis of attention, psychomotor speed, executive function (TMT B, and Stroop Interference Score), working memory or verbal memory (VLMT Trial 5–7 and recognition—mistake) did not show a significant correlation to current PA and SB.

After adjusting for covariates (age, BMI, education, gender) the association between executive control (TMT ratio score B/A) and LIPA remained significant (*r* = −0.339, *p* = 0.015, df = 49).

The correlation between verbal memory (VLMT *Σ* Trial 1–5) and TAC (*r* = 0.114, *p* = 0.429, df = 48), LIPA (*r* = 065, *p* = 0.653, df = 48) and SB (*r* = −161, *p* = 0.263, df = 48) was no longer significant after controlling for covariates. Details of bivariate and partial correlations between CF and current PA are shown in Table 2.

**Table 2:** Bivariate Spearman and partial correlations between cognitive performance and current physical activity.

	**Current physical activity**			
	**TAC (min/day)**	**MVPA (min/week)**	**LIPA (min/week)**	**SB (min/week)**
**Attention and psychomotor speed**				
Trail Making Test-A (in sec); *n* = 55	−0.173	−0.228	0.180	0.078
**Executive function**				
Trail Making Test-B (in sec); *n* = 55	−0.217	−0.198	−0.112	0.172
TMT-B/TMT-A (in sec); *n* = 55	−0.056	0.031	−0.359^**^	−0.080
Adjusted for covariates; df = 49	0.147	0.184	−0.339^*^	−0.059
Stroop Interference Score (in sec); *n* = 52	−0.026	0.059	−0.185	0.232
**Working memory**				
Digit Span forward (in points); *n* = 56	−0.044	0.100	−0.184	−0.117
Digi Span backward (in points); *n* = 56	0.215	0.147	0.052	−0.216
**Verbal memory**				
VLMT Σ Trial 1–5 (in items); *n* = 56	0.294^*^	0.215	0.767^*^	−0.388^**^
Adjusted for covariates; df = 48	0.114	0.076	0.065	−0.161
VLMT Trial 5–7 (in items); *n* = 56	0.018	0.037	−0.171	0.058
VLMT recognition—mistake (in items); n = 54	0.176	0.139	0.240	−0.225

*Each field indicates correlation coefficient r value. Level of significance is*

* *p < 0.05 and*

** *p < 0.01 indicated by bold font*.

### Relationship Between Cognitive Function and Physical Performance

Attention and psychomotor speed (TMT A) showed a significant association with PP (VO_2peak_: *r* = −0.362, *p* = 0.007; max. workload: *r* = −0.354, *p* = 0.008; relative max. workload: *r* = −0.285, *p* = 0.035; max. HR: *r* = −0.351, *p* = 0.009; HRR: *r* = −0.303, *p* = 0.025) in Spearman correlations. As a reminder, small values in TMT indicate a better performance. Executive function (TMT B) was significantly associated with VO_2peak_ (*r* = −0.330, *p* = 0.014), relative max. workload (*r* = −0.266, *p* = 0.050), max. HR (*r* = −0.372, *p* = 0.005) and HRR (*r* = −0.300, *p* = 0.026). Moreover, working memory was significantly associated with VO_2peak_ (DSF: *r* = 0.321, *p* = 0.016; DSB: *r* = 0.393, *p* = 0.003), max. workload (DSF: *r* = 0.401, *p* = 0.002; DSB: *r* = 0.377, *p* = 0.004), as well as relative max. workload (DSF: *r* = 0.354, *p* = 0.007; DSB: *r* = 0.391, *p* = 0.003). In addition, verbal memory (VLMT recognition—mistakes) correlated significantly with max. HR (*r* = 0.274, *p* = 0.045) and HRR (*r* = 0.327, *p* = 0.016). Further analysis of executive function (TMT B/A and Stroop Interference Score) or verbal memory (VLMT *Σ* Trial 1–5 and recognition—mistake) did not show a significant correlation to PP.

After adjusting for covariates (age, BMI, education, gender) attention and psychomotor speed remained significantly associated with VO_2peak_: (*r* = −0.337, *p* = 0.015, df = 49) and max. absolute workload (*r* = −0.286, *p* = 0.042, df = 49). Moreover, working memory correlated beneficially with max. workload (DSF: *r* = 0.329, *p* = 0.017, df = 50; DSB: *r* = 0.274, *p* = 0.049, df = 50).

The correlation between attention, psychomotor speed, and relative max. workload (*r* = −0.263, *p* = 0.062, df = 49), max. HR (*r* = −0.263, *p* = 0.062, df = 49) and HRR (*r* = −0.196, *p* = 0.167, df = 49) was no longer significant. Executive function (TMT B) and VO_2peak_ (*r* = −0.195, *p* = 0.171, df = 49), max. workload (*r* = −0.193, *p* = 0.174, df = 49), relative max. workload (*r* = −0.155, *p* = 0.279, df = 49), max. HR (*r* = −0.128, *p* = 0.371, df = 49) and HRR (*r* = −0.068, *p* = 0.634, df = 49) were not associated anymore. Furthermore, there was no correlation between working memory and VO_2peak_ (DSF: *r* = 0.248, *p* = 0.076, df = 50; DSB: *r* = 0.245, *p* = 0.076, df = 50), relative max. workload (DSF: *r* = 0.251, *p* = 0.073, df = 50; DSB: *r* = 0.235, *p* = 0.094, df = 50) as well as between verbal memory (VLMT recognition—mistakes), max. HR (*r* = 0.193, *p* = 0.178, df = 48) and HRR (*r* = 0.228, *p* = 0.111, df = 48). Details of bivariate and partial correlations between cognitive performance and PP are presented in Table 3.

**Table 3:** Bivariate Spearman and partial correlation between cognitive performance and physical performance.

***N* = 56**	**Physical performance**				
	**Peak oxygen uptake**	**Max. workload**	**Relative max. workload**	**Max. HR**	**HRR**
	**(ml/kg/min)**	**(Watts)**	**(Watts/kg)**	**(bpm)**	**(bpm)**
**Attention and psychomotor speed**					
Trail Making Test-A (in sec); *n* = 55	−0.362^**^	−0.354^**^	−0.285^*^	−0.351^**^	−0.303^*^
Adjusted for covariates; df = 49	−0.337^*^	−0.286^*^	−0.263	−0.263	−0.196
**Executive function**					
Trail Making Test-B (in sec); *n* = 55	−0.330^*^	−0.259	−0.266^*^	−0.372^**^	−0.300^*^
Adjusted for covariates; df = 49	−0.195	−0.193	−0.155	−0.128	−0.068
TMT-B/TMT-A (in sec); *n* = 55	0.043	0.098	0.019	0.037	0.061
Stroop Interference Score* (in sec); *n* = 52	−0.016	0.010	0.084	−0.053	−0.003
**Working memory**					
Digit Span forward (in points); *n* = 56	0.321^*^	0.401^**^	0.354^*^	0.169	0.155
Adjusted for covariates; df = 50	0.248	0.329^*^	0.251	0.156	0.115
Digi Span backward (in points); *n* = 56	0.393^**^	0.377^**^	0.391^**^	0.154	0.088
Adjusted for covariates; df = 50	0.245	0.274^*^	0.235	0.110	0.014
**Verbal Memory**					
VLMT Σ Trial 1–5 (in items); *n* = 56	0.183	0.106	0.102	0.204	0.125
VLMT Trial 5−7 (in items); *n* = 56	0.135	0.237	0.125	−0.118	−0.133
VLMT recognition—mistakes (in items); n = 54	0.115	0.023	0.082	0.274^*^	0.327^*^
Adjusted for covariates; df = 48	0.008	0.012	0.000	0.193	0.228

*Cognitive performance was assessed as attention and psychomotor speed (Trail Making Test (TMT) Part A in sec), executive function (TMT Part B in sec, ratio score B/A in sec, Stroop Interference Score in sec), working memory (digit span forward and backward in points) and verbal memory (Verbal Learning and Memory Test, VLMT: Total Learning Performance (Σ Trial 1–5), loss after delay (Trial 5–7), Correct Recognition Performance (recognition—mistakes) in items). The physical performance was analyzed as peak oxygen uptake (ml/kg/min), maximal workload (Watt), relative maximal workload (Watt to bodyweight ratio), maximal heart rate (HR, bpm) and heart rate reserve (HRR, maximal heart rate—resting heart rate, bpm)*.

*Each field indicates correlation coefficient r value. Level of significance is*

* *p < 0.05 and*

** *p < 0.01 indicated by bold font*.

## Discussion

The present study analyzes the association of domain specific CF (attention and psychomotor speed, executive function, working memory and verbal memory) with accelerometer-based PA and SB as well as cardiopulmonary exercise testing-based performance measures in healthy older adults.

We found a significant association of executive control and LIPA. Another major finding of our study is the relationship of attention and psychomotor speed with aerobic capacity and maximal power output during exercise testing. Our data underline the need for rigorous control of confounders as the associations between PP and executive function or memory were influenced by age, sex, education, BMI. Likewise, associations of LIPA and SB with memory performance seem to be driven by the confounding influence of age, sex, education, and BMI.

### Physical Activity and Cognitive Function

The present study found no relationship between attention, psychomotor speed, and PA. These findings are in line with other studies (Kerr et al., 2013; Lambiase et al., 2014; Johnson et al., 2016; Wilckens et al., 2018). Johnson et al. (2016) analyzed PA and CF in 188 community-dwelling cognitively healthy older adults. When controlling for age, smoking history, alcohol intake, educational achievement and neuropsychological functioning, higher levels of current PA were not associated with attention and psychomotor speed (TMT A) (Johnson et al., 2016). In line with our results, these findings underline the relevance of sufficient confounder analysis especially in cross-sectional designs. Furthermore, the study by Kerr et al. (2013) also supports our results. They did not find a correlation between attention, psychomotor speed, and PA after adjusting for covariates in older adults.

To our knowledge no previous study has examined the relationship between PA and executive control function (TMT ratio score B/A) in older adults. Furthermore, earlier studies on the association between overall CF and PA used activity measures such as TAC and did not analyze the impact of PA intensity. A major finding of our analysis was a significant small association of executive control function (TMT ratio score B/A) and LIPA. However, we did not find a relationship with shifting and task switching capabilities (TMT-B). This is in accordance to other studies (Kerr et al., 2013; Lambiase et al., 2014). Kerr et al. (2013) could show a significant correlation between LIPA and executive function (TMT-B, TMT B-A) but significance was absent after adjustment for covariates. Johnson et al. (2016) could show a relation between shifting and task switching capabilities (TMT-B) and LIPA in older healthy adults. In contrast to some earlier findings of Kerr et al. (2013), our study and Johnson et al. (2016) showed no correlation between PA and MVPA. Since 2020, LIPA is implemented in current PA guidelines (Bull et al., 2020). But as these results illustrate, it is necessary to invest more research in the field of LIPA and CF.

Furthermore, our study could not find a relationship between Stroop Interference Score and current PA, which is in line with Wilckens et al. (2018). However, Wilbur et al. (2012) could show a beneficial association between executive function (Stroop-Test) and LIPA in older (age and cognitive status) Latinos. In addition, in the study of Boucard et al. (2012) PA was assessed via accelerometer and participants where split into a sedentary and an active group. The active group performed significantly better in the inhibition task composed of three different tasks (one was the Stroop Test). However, both studies did not adjust for covariates. These results also demonstrate the importance of taking confounding factors into account, especially in cross-sectional designs.

Our study could not find a relationship between working memory and LIPA or MVPA. To our knowledge no previous study has examined the relationship of objectively measured LIPA and MVPA with auditory working memory (digit span) in older adults. Brown et al. (2012) assessed auditory working memory via digit spans and PA via accelerometry but they did not analyze LIPA or MVPA. They used peak counts score, which is a measure of intensity, and reflects the highest intensity (peak count) recorded for each day. They found a beneficial association between peak counts and working memory and therefore indicate the relevance of high intensity activities. Other studies have assessed visual working memory (Wanigatunga et al., 2018; Wilckens et al., 2018) and came to the same results as we did. Wanigatunga et al. (2018) did not find any association between visual working memory and different bout length of PA. These findings indicate that light and moderate PA have no effect on CF whereas vigorous PA might have.

The present study could show an association between verbal memory and TAC, LIPA, and SB in the unadjusted correlation analysis. However, significance was lost after adjusting for covariates (age, sex, education, BMI). This is probably due to the influence of age on both parameters and the influence of sex on PA. Our results concerning verbal memory function are in line with two studies with older Latinos (Wilbur et al., 2012; Vásquez et al., 2017). In contrast, Zhu et al. (2015, 2017) could show a beneficial association between memory and MVPA in older adults. These inconsistent results are probably due to the different cut points to classify the intensities of PA (SB 0–49 cpm, LIPA 50–1,064 cpm, and MVPA >1,065 cpm in Zhu et al. (2015, 2017) versus SB ≤ 99 cpm, LIPA 100–1,951 cpm, MVPA ≥ 1,952 cpm in Freedson et al. (1998). Wanigatunga et al. (2018) investigated the effect of different bout length of PA and could show a beneficial relationship between memory and longer bout lengths of ≥5 min. Therefore, not only the intensity but also the length of PA could be important to explain the relationship between PA and verbal memory.

### Sedentary Behavior and Cognitive Function

Our study could not show a relationship between SB and any of the subdomains of CF. Our findings are in contrast to the study of Kesse-Guyot et al. (2012) and Hamer and Stamatakis (2014) which found that regular computer use is beneficially associated with verbal memory whereas watching television has a detrimental relation with executive function. One explanation could be that we assessed total SB and did not distinguish between different activities. It is possible that certain sedentary behaviors have a negative and others a positive effect on CF. Another explanation is that Kesse-Guyot et al. (2012) and Hamer and Stamatakis (2014) measured SB with self-reported questionnaires, which might be biased (Reilly et al., 2008; Dall et al., 2017), whereas we used objective data from the accelerometer. However, two studies which assessed SB with the accelerometer ActivPAL and CF via neuropsychological test batteries could not find any evidence that objectively measured sedentary time in older adults is associated with measures of cognitive functioning in older age (Lord et al., 2011; Cukić et al., 2018). The study by Rosenberg et al. (2016) support the results described above. They could show that there is a beneficial relation between self-reported SB and attention and psychomotor speed (measured with the TMT-A) but no significant association between objectively measured SB and executive function, attention, and psychomotor speed (measured with the TMT).

In addition, our results are consistent with Rosenberg et al. (2016) who used the same outcome measures, and did not find any relationship between objectively measured SB and executive function and attention assessed with the TMT A and B. Taken together, SB does not seem to be detrimental for maintained CF.

### Physical Performance and Cognitive Function

The current study showed a beneficial correlation between attention, psychomotor speed, and PP. After adjusting for covariates, significance for relative max. workload, max. HR as well as HRR was lost but there was still a trend with an alpha level under 0.10. More importantly, our study could show a beneficial small relationship between attention, psychomotor speed, and aerobic capacity as well as maximal power output during cardiopulmonary exercise testing.

These findings are in accordance to those of Newson and Kemps (2006), who compared young (18–30 years), young-old (65–74 years), middle-old (75–84 years) and old-old adults (85–92 years) in a cross-sectional study investigating the relationship between cardio respiratory fitness (VO_2max_) and attention (parts from the Stroop Test and Map Search Test). Our results are however not in line with Burns et al. (2008) who found a relationship between attention and aerobic capacity but after adjusting for age, significance was lost. Billinger et al. (2017) also did not find any relationship. In addition, Winker et al. (2010) failed to find a beneficial association between attention and individual physical working capacity. This is probably due to differences in study design. Winker et al. (2010) investigated elderly marathon runners or bicyclists and compared them to an inactive control group. Our findings outline the relevance of maintaining maximal PP in older adults for coping cognitively and physically challenging tasks during everyday life. Especially for daily living, older adults require their maximal PP, for example, if they need to cross a highly frequented road or take flights of stairs.

Our study could show a beneficial relationship between shifting and task switching capabilities (TMT B) and different parameters of PP, but significance was lost after adjustment for covariates. This is in line with recent studies (Burns et al., 2008; Winker et al., 2010; Billinger et al., 2017). Furthermore, we did not find a beneficial relationship between executive control function performance (TMT-B/TMT-A) and different parameters of PP, which is accordance to Winker et al. (2010). In addition, there was no beneficial association between Stroop Interference Score and the parameters of PP. These findings are in line with other studies (Burns et al., 2008; Winker et al., 2010; Abou-Dest et al., 2012). Our findings are supported by Abou-Dest et al. (2012) who analyzed executive function accessed via seven different tests. Two tasks (updating of verbal information's and global switch cost) were significant correlated with VO_2peak_ level, but executive function assessed via Stroop Test was not associated. In contrast, Weinstein et al. (2012) could show a beneficial relation between Stroop Interference Score and VO_2peak_ in older adults. However, participants in that study were considerably younger (mean 66.6, range 58–81 years) than ours (76 ± 7 years). VO_2peak_ might be a predictor for executive function in middle-aged adults but not in older adults, or other influencing factors (such as diet or social activities) may exert more impact over the lifespan.

We could show a beneficial relation between verbal memory (VLMT recognition—mistakes) and max. HR and HRR. After adjusting for covariates significance was lost. Our results are in accordance to other studies (Newson and Kemps, 2006; Burns et al., 2008; Winker et al., 2010; Billinger et al., 2017).

Our results showed a beneficial association between working memory and max. workload but no relationship with other parameters of PP. Burns et al. (2008) and Erickson et al. (2012) used the same cognitive assessment methods as we did and did not find a relationship between working memory and VO_2peak_. We are not aware of any previous study which investigated the effect of other parameters of PP (max. workload, relative max. workload, max. HR and HRR) with working memory. These results indicate that aerobic metabolism performance might not be the only explanatory approach for the relationship between PP and working memory, and anaerobic metabolism, muscle strength and coordination may also impact working memory.

### Physiological Mechanisms

The underlying mechanisms behind the protective effects of PA on CF are not fully understood. Promising explanatory approaches in human studies are centered on endurance training induced changes in neurotransmitter concentrations such as BDNF and increased regional cerebral blood flow, which could explain the improvement of CF (Erickson et al., 2011). Others focus on altered brain metabolism and hypothesize associations of exercise with cortical acetylcholine uptake and increased dopamine receptor density in the brain based on murine models (Fordyce and Farrar, 1991). Furthermore, an increase in the efficiency of neurotransmitters after exercise is discussed (McAuley et al., 2004). Taken together, these changes might promote neurogenesis, angiogenesis and synaptogenesis (Erickson et al., 2011) and thus could lead to increased brain plasticity in crucial brain areas, like the hippocampus (Cotman et al., 2007).

### Strengths and Limitations

Our study results are limited by its cross-sectional design, which precludes the establishment of causation. In addition, we cannot make any statements about clinical relevance based on our statistics. Another limitation is that the sample is small and non-representative of a broader population. A strength is that we incorporate an analysis of covariates (age, sex, education, and BMI). Most of our participants were highly educated with a mean of 12.6 education years but relatively homogenous with a standard deviation of 3.5 years. Therefore, lower educated older adults might be underrepresented in our study. Furthermore, another strength is that we included participants with higher age (65–90 years). Subjects were examined to guarantee a high quality of outcomes. We assessed PA with accelerometers and although it is an objective method, it still has some limitations. The uniaxial accelerometer was worn on the hip. Therefore, the disadvantage of the accelerometer is that it is underestimating PA intensity in which the hips have limited movement, for example in bicycling, doing chair gymnastics or other activities where only the upper body is moving (Kerr et al., 2013). Furthermore, it must not be exposed to wet conditions. Hence, participants could not wear the accelerometer during swimming or aqua fitness. However, we analyzed the PA in different intensities and the range of the PA profile of our participants was heterogonous. Furthermore, a strength of our study is that we assessed physical fitness as cardiorespiratory fitness, anaerobic and aerobic capacity via cardiopulmonary exercise testing. This method is the most recommended for these parameters (Young et al., 2015). Another strength of the study is that we analyzed the cognitive performance in four different domains which are of interest when examining the relationship of PA and PP (Smith et al., 2010).

## Conclusion

The present study analyzes the association of domain specific CF (attention and psychomotor speed, executive function, working memory and verbal memory) with accelerometer-based PA and SB as well as objectively measured PP in healthy older adults. The major finding of our study that the executive control function is significant associated with LIPA supports the implementation of LIPA in the current PA guidelines (Bull et al., 2020). Another major finding of our study is the relationship between attention, psychomotor speed, and aerobic capacity as well as maximal power output during cardiopulmonary exercise testing. Moreover, our study could show a significant association between working memory capacity and maximal power output during cardiopulmonary exercise testing. The findings of the present study indicate that cardiorespiratory fitness accounts for single-component processing resources such as attention, processing speed and working memory but not or to a lesser extent for multi-component higher-order CF such as executive function and verbal memory. These CF are very important for daily living because they are involved in almost every task we accomplish. Therefore, it is important to maintain or even improve cardiorespiratory fitness also in older adults. In future studies it is important to investigate different cognitive domains.

## Data Availability Statement

The raw data supporting the conclusions of this article will be made available by the authors, without undue reservation.

## Ethics Statement

The studies involving human participants were reviewed and approved by Medical Faculty of Goethe University Frankfurt (reference 107/13). The patients/participants provided their written informed consent to participate in this study.

## Author Contributions

SG, TE, JF, EF, SM, and UP collected the data. TE, JF, EF, SM, UP, LV, JP, and WB designed the study. SG and TE analyzed the data and wrote the manuscript. All authors edited the manuscript.

## Funding

This work was supported by the Else-Kröner-Fresenius-Foundation, the Cronstetten Foundation, and the Familie Schambach Foundation (no grant numbers available), all of them German non-profit foundations guaranteeing independency of research. We acknowledge support for the publication costs by the Open Access Publication Fund of Bielefeld University.

## Conflict of Interest

The authors declare that the research was conducted in the absence of any commercial or financial relationships that could be construed as a potential conflict of interest.

## Publisher's Note

All claims expressed in this article are solely those of the authors and do not necessarily represent those of their affiliated organizations, or those of the publisher, the editors and the reviewers. Any product that may be evaluated in this article, or claim that may be made by its manufacturer, is not guaranteed or endorsed by the publisher.
